# Dietary Carvacrol Attenuates Cyclophosphamide‐Induced Neurotoxicity: Implications for Food‐Derived Neuroprotection and Molecular Mechanisms

**DOI:** 10.1002/fsn3.70734

**Published:** 2025-08-07

**Authors:** Hamit Emre Kızıl

**Affiliations:** ^1^ Department of Medical Services and Techniques, Vocational School of Health Services Bayburt University Bayburt Türkiye

**Keywords:** apoptosis, autophagy, carvacrol, cyclophosphamide, neuroinflammation, neurotoxicity, oxidative stress

## Abstract

Carvacrol (CRV) is a phenolic monoterpene abundant in culinary herbs such as oregano and thyme and is well known for its potent antioxidant, anti‐inflammatory, and neuroprotective properties. This study investigated the ability of CRV to counteract neurotoxicity induced by cyclophosphamide (CP), a widely used antineoplastic agent. Male *Wistar albino* rats were divided into five groups and received CP and/or CRV treatments. Neurotoxicity and neuroprotection were evaluated through biochemical assays, real‐time PCR, histopathological and immunohistochemical analyses, and behavioral testing (Morris Water Maze). CP administration led to significant increases in oxidative stress markers, disruption of antioxidant enzyme activities, upregulation of inflammatory mediators (NF‐κB, TNF‐α, iNOS), dysregulation of apoptotic regulators (increased Bax and Casp‐3, decreased Bcl‐2), alterations in autophagy markers (Beclin‐1, LC3A, LC3B), and suppression of Notch1/Hes1 signaling. Histopathological analyses revealed neuronal degeneration, vascular hyperemia, and increased GFAP and 8‐OHdG expression in brain tissue. CRV treatment, particularly at higher doses, effectively mitigated these biochemical, molecular, and histological alterations. Notably, CRV administration preserved spatial learning and memory function in CP‐treated rats, as demonstrated by the Morris Water Maze test, indicating functional neuroprotection. These findings highlight the multifaceted neuroprotective mechanisms of CRV and suggest its potential as a food‐derived bioactive compound for development into functional foods or dietary supplements to improve the quality of life for cancer patients undergoing chemotherapy.

## Graphical abstract

Carvacrol, a compound found in herbs like oregano and thyme, shows promise in protecting against cyclophosphamide‐induced neurotoxicity in rats by reducing oxidative stress, inflammation, and cell death, and by preserving cognitive function.

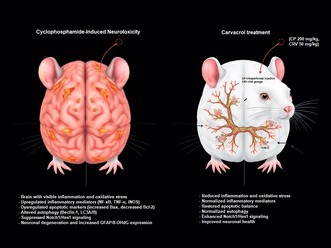

## 1 Introduction

Carvacrol (CRV), a monoterpenic phenol and the major component of essential oils derived from oregano ( *Origanum vulgare* ), thyme ( *Thymus vulgaris* ), and other aromatic plants, has garnered significant scientific attention due to its diverse biological activities (Imran et al. 2022). CRV, which has been approved by the FDA as a food additive and listed as a flavoring agent by the Council of Europe (Mączka et al. 2023), demonstrates a wide spectrum of biological activities including antimicrobial (Mauriello et al. 2021; Memar et al. 2017), anti‐acetylcholinesterase (Kurt et al. 2017), antiproliferative (Chen and Fong 2022), antidepressant (Vilmosh et al. 2022), hepatoprotective (Bakır et al. 2016), antidiabetic (Li et al. 2020), and antiparkinsonian (Manouchehrabadi et al. 2020) effects, making it not only a valuable pharmacological agent but also an important bioactive compound in food science and human nutrition.

Turkey is recognized as a global leader in the production and export of oregano and oregano oil, both of which are rich sources of carvacrol. Versatile biological activities enable CRV to be used not only in medicine but also as a food additive, in apiculture, and for the management of gastrointestinal disorders (Can Baser 2008). The broad spectrum of biological effects exhibited by CRV is closely related to its molecular structure and biosynthetic pathway. Biosynthesized via the mevalonate pathway, carvacrol has been shown to induce apoptosis, inhibit metastasis, and suppress various signaling pathways in cancer cells. In addition, it has been reported to improve liver function, regulate insulin and glucose levels, and exert potent antimicrobial effects against a wide range of pathogens (Imran et al. 2022).

Such a wide range of applications has driven the development of new technologies to enhance the bioavailability and efficacy of CRV. In particular, polylactic acid/carvacrol (PLA/CAR)‐based materials have emerged as promising candidates for use in food packaging, medical device coatings, and drug delivery systems due to their environmentally friendly and biocompatible properties (Scaffaro et al. 2020). Carvacrol has also demonstrated potential in preventing biofilm contamination in the food industry by inhibiting biofilm formation and virulence factors of *Listeria monocytogenes* (Li et al. 2025). In the food sector, antimicrobial compounds such as carvacrol are incorporated into various polymer matrices to develop antimicrobial packaging systems aimed at controlling microbial activity and enhancing product safety (Ramos et al. 2025). Furthermore, emulsions containing carvacrol and astaxanthin have been shown to improve microbial stability and physicochemical properties in nitrite‐free meat products, enhancing microbiological safety and potentially serving as nitrite alternatives (Kim et al. 2025). The application of carvacrol to fruits has been reported to delay ripening, increase antioxidant activity, and extend shelf life (Wang et al. 2025). In photodynamic inactivation (PDI) systems, carvacrol combined with LED illumination effectively eliminates pathogens such as Salmonella from food surfaces without compromising product quality (Keyvan et al. 2025).

The pharmacokinetic properties of CRV are also central to its biological effects. When administered orally, 30% of the dose remains in the gastrointestinal system, while 25% is excreted in urine after 22 h. Due to its small molecular size and fat‐soluble nature, CRV readily crosses the blood–brain barrier, allowing effective access to the central nervous system (Abbasloo et al. 2023). CRV exhibits remarkable antioxidant properties by directly scavenging free radicals and enhancing cellular antioxidant defense mechanisms (Suntres et al. 2015). Its bioavailability can be enhanced through alginate‐whey protein microencapsulation (Wang et al. 2009). CRV is metabolized through two main pathways: in Phase II metabolism, the phenolic group is conjugated with glucuronic acid and sulfate, while in Phase I metabolism, terminal methyl groups are oxidized to primary alcohols (Dong et al. 2012).

These pharmacokinetic advantages have encouraged research into the effects of CRV on the central nervous system. CRV demonstrates potent anti‐inflammatory effects through modulation of pro‐inflammatory mediators and has shown neuroprotective potential in various experimental models (Zamanian et al. 2021) It has the capacity to enhance memory and cognition by modulating oxidative stress and inflammation in neurodegenerative conditions, as well as reducing reactive oxygen species and proinflammatory cytokine levels in neurological disorders (Azizi et al. 2022; Javed et al. 2023). Recent studies have highlighted CRV's ability to cross the blood–brain barrier effectively, making it particularly valuable for treating neurological conditions (Asle‐Rousta 2025). Previous studies have demonstrated that CRV treatment eliminated the harmful effects of LPS and improved learning and memory in LPS‐exposed rats, with these effects being attributed to its anti‐inflammatory and antioxidant properties in the brain, though the exact mechanisms require further investigation (Hakimi et al. 2020). These findings support CRV's potential as a neuroprotective agent.

Recent research has further expanded the pharmacological profile of CRV beyond its traditional antioxidant and anti‐inflammatory actions. Notably, CRV has been shown to modulate neurotrophic factors such as brain‐derived neurotrophic factor (BDNF) and to influence synaptic plasticity, which are critical for learning and memory processes (Gao et al. 2018). In experimental models of neurodegeneration, CRV administration has resulted in the upregulation of neuroprotective genes and the downregulation of pro‐apoptotic pathways, suggesting a broader regulatory role in neuronal survival (Bağcı et al. 2025). Moreover, CRV has demonstrated the ability to attenuate blood–brain barrier disruption and reduce neurovascular inflammation, which are increasingly recognized as key contributors to chemotherapy‐induced cognitive impairment. Recent in vivo imaging studies have confirmed that CRV can accumulate in brain tissue at pharmacologically relevant concentrations, supporting its translational potential for central nervous system disorders (Abbasloo et al. 2023). Emerging evidence also suggests that CRV may interact with the gut‐brain axis, modulating gut microbiota composition and reducing systemic inflammation, which could indirectly contribute to its neuroprotective effects (Mooyottu et al. 2017).

Furthermore, carvacrol has demonstrated anti‐inflammatory and antioxidant effects in inflammatory diseases such as endometriosis, with animal and cell culture studies showing that it reduces lesion size, increases T cell populations, and modulates the expression of relevant genes (Jang et al. 2025). In addition, carvacrol‐mediated synthesis of zinc oxide quantum dots (CVC‐ZnO QDs) has been reported to suppress tumor growth in breast cancer cells by modulating the PI3K/AKT/mTOR pathway, inducing apoptosis, and promoting oxidative stress (Srinivasan and Namasivayam 2025).

Taken together, these findings indicate that CRV is a versatile and promising bioactive agent in both the food industry and medicine. In particular, in cases of central nervous system damage such as chemotherapy‐induced neurotoxicity, CRV may provide functional improvement through its antioxidant, anti‐inflammatory, and neuroprotective effects. In this study, the protective effects of CRV against cyclophosphamide‐induced neurotoxicity were comprehensively evaluated, and the findings support the potential of CRV to be developed as a functional food or dietary supplement.

Chemotherapy‐induced neurotoxicity represents a significant global healthcare challenge, affecting 20%–50% of cancer patients at standard doses and nearly all patients at high doses, substantially impacting their quality of life (Yu et al. 2024). Among chemotherapeutic agents, Cyclophosphamide (CP), first introduced in 1958 and FDA‐approved, remains a cornerstone in cancer treatment, widely employed in the treatment of hematological malignancies, solid tumors, and autoimmune diseases (Famurewa et al. 2022). Recent epidemiological studies indicate that approximately 30%–40% of patients receiving CP‐based chemotherapy experience neurotoxic complications, highlighting an urgent need for protective interventions (Staff et al. 2017). CP, as a prodrug, undergoes complex biotransformation in the liver through cytochrome P‐450 enzymes, forming hydroxy‐cyclophosphamide and subsequently aldophosphamide, which cleaves into phosphoramide mustard and acrolein (Was et al. 2022). The anticancer mechanism primarily involves phosphoramide mustard (Povirk and Shuker 1994), which targets DNA by forming covalent adducts and creating interstrand crosslinks at the guanine N‐7 position, disrupting DNA structure, hindering repair mechanisms, and inducing apoptosis (Emadi et al. 2009). However, CP's cytotoxic effects extend beyond cancer cells, damaging healthy organ cells and resulting in toxicity‐related side effects (Famurewa et al. 2023). The neurotoxic effects of CP manifest through complex and multifactorial mechanisms, affecting both central and peripheral nervous systems. Among its toxic metabolites, acrolein, being highly lipophilic, easily crosses the blood–brain barrier (BBB) and contributes significantly to neurotoxicity (Mishra et al. 2022; Moghe et al. 2015). These metabolites induce oxidative stress through excessive ROS production, cause mitochondrial dysfunction, and trigger apoptotic cell death (Crouch et al. 2017). Additionally, they exacerbate neuroinflammation through microglial activation and intensify neuronal damage via glutamate excitotoxicity (Ibrahim et al. 2024; Ren et al. 2019). Indeed, oxidative stress and inflammation represent critical pathological mechanisms underlying the initiation and progression of neurotoxicity (Chitnis and Weiner 2017). While the central nervous system (CNS) effects of CP have been well documented, the full spectrum of its peripheral neurotoxic effects and underlying mechanisms remains inadequately explored (Singh and Kumar 2019). This knowledge gap is particularly significant as peripheral neurotoxicity manifests as neuropathic pain, chemobrain, and enteric neuropathy, substantially impacting patients' quality of life and potentially limiting treatment efficacy (Ogino and Tadi 2024). Natural compounds, particularly those with established antioxidant and anti‐inflammatory properties, have emerged as promising therapeutic candidates for preventing chemotherapy‐induced neurotoxicity (Waseem and Parvez 2016).

Numerous studies have investigated the protective effects of natural compounds in preventing or alleviating chemotherapy‐induced neurotoxicity. In particular, the natural alkaloid berberine (BER) has been reported to reduce cognitive impairment and exert neuroprotective effects in rodents treated with doxorubicin (DOX), an effect attributed to the restoration of metabolic balance via increased SIRT1 protein expression (Shaker et al. 2021). Additionally, γ‐glutamyl cysteine ethyl ester, a glutathione precursor, has been shown to elevate glutathione levels and reduce oxidative damage in brain tissue following adriamycin (doxorubicin) administration (Joshi et al. 2007). Furthermore, cognitive deficits induced by oxaliplatin and 5‐fluorouracil have been ameliorated by physical activity (Fardell et al. 2012). Consistent with these findings, recent studies have demonstrated that phytochemicals such as curcumin, resveratrol, and thymoquinone can attenuate chemotherapy‐induced neurotoxicity by modulating oxidative stress and neuroinflammation. For instance, curcumin and resveratrol have shown protective effects against cisplatin‐ and paclitaxel‐induced neurotoxicity in preclinical models, while thymoquinone has been reported to ameliorate cyclophosphamide‐induced neuronal damage (Chung and Kim 2022). Similarly, silymarin has demonstrated neuroprotective effects against docetaxel‐induced central and peripheral neurotoxicity by enhancing antioxidant defenses and suppressing inflammation and apoptosis in experimental models (Yardım et al. 2021). Additionally, morin, a natural flavonoid, has been shown to exert a chemoprotective effect against ifosfamide‐induced acute neurotoxicity by modulating oxidative stress, inflammation, and apoptotic pathways in experimental models (Çelik et al. 2020). These findings collectively suggest that natural compounds and lifestyle interventions may offer potential benefits in mitigating chemotherapy‐induced neurotoxicity. These findings highlight the therapeutic potential of natural compounds and provide a rationale for further investigation of agents such as CRV. Despite the well‐documented biological activities of CRV, there is no clear evidence in the literature regarding its protective effects against CP‐induced neurotoxicity.

Therefore, the aim of this study was to investigate the protective effects of CRV against CP‐induced neurotoxicity by examining its impact on oxidative stress, inflammatory mediators (NF‐κB, TNF‐α, iNOS), apoptotic regulators (Bax, Casp‐3, Bcl‐2), autophagy markers (Beclin‐1, LC3A, LC3B), and Notch1/Hes1 signaling pathway through biochemical, molecular, histopathological, and behavioral approaches.

## 2 Materials and Methods

### 2.1 Chemical Compounds and Reagents

The primary compounds utilized in this investigation, including CP (CP, CAS No.: 50‐18‐0, purity 99%) and CRV (CRV, CAS No.: 499‐75‐2, purity 97%), were procured at analytical grade purity from Sigma Chemical Co. (St. Louis, US).

### 2.2 Experimental Animals and Study Design

The experimental protocol was executed using 35 mature male *Wistar albino* rats, aged 3 months and weighing between 220 and 250 g. The animals were housed under controlled environmental conditions at KONUDAM Experimental Medicine Application and Research Center (Konya/Türkiye), with consistent temperature regulation, relative humidity maintained at 50% ± 5%, and a standardized 12‐h light/dark cycle. Throughout the experimental period, all animals had unrestricted access to standard laboratory chow and fresh water. The study was designed as a randomized controlled, single‐blind experiment consisting of five independent groups. One‐way analysis of variance (ANOVA) was used to determine the sample size. In accordance with animal welfare principles and considering similar studies in the literature, it was decided to work with a total of 35 rats, with seven animals per group. Random assignment to groups was performed using a five‐block randomization method via Random Allocation Software (RAS). The dosages used in this study were selected based on previous research demonstrating efficacy in similar experimental models, with the CP dose determined according to Famurewa et al. (2023), who established its neurotoxic effects at this concentration, and the CRV doses selected based on the findings of Kandemir et al. (2021) and Şimşek et al. (2024) who utilized two different concentrations of CRV demonstrating optimal neuroprotective properties in rodent models. The experimental groups are as follows:

Control Group: Rats were administered intraperitoneal saline for 7 days.

CRV Group: Rats were administered oral CRV (50 mg/kg/day) for 7 days.

CP Group: Rats were administered a single intraperitoneal injection of CP (200 mg/kg) on day 7.

CP + CRV‐25 Group: Rats were administered oral CRV (25 mg/kg/day) for 7 days and a single intraperitoneal injection of CP (200 mg/kg) on day 7.

CP + CRV‐50 Group: Rats were administered oral CRV (50 mg/kg/day) for 7 days and a single intraperitoneal injection of CP (200 mg/kg) on day 7.

### 2.3 Cognitive Deficit Assessment Through Morris Water Maze

The Morris Water Maze (MWM) test was employed to evaluate spatial learning and memory capabilities, adapting the protocol described by Topuz et al. (2020). The apparatus consisted of a circular water tank (150 cm in diameter, 45 cm in depth) filled with water. The tank was divided into four equal quadrants, with a platform (10 × 10 cm) positioned in the center of the southwest quadrant. The platform was submerged 2 cm below the water surface, and non‐toxic black paint was used to render the water opaque. The testing room was maintained under constant illumination, and various visual cues and objects were placed on the surrounding walls to facilitate spatial navigation. All swimming trials were recorded using a ceiling‐mounted video camera. The experimental procedure comprised two phases: acquisition training and probe trial. During the acquisition phase (days 8–10), each animal completed four daily trials for five consecutive days. For each trial, rats were placed into the pool from different quadrants and allowed to swim for 60 s. Upon locating the platform, rats were permitted to remain on it for 15 s before being removed. If an animal failed to find the platform within 60 s, it was gently guided to the platform and allowed to stay for 15 s. The probe trial was conducted 24 h after the final acquisition trial (day 12), during which the platform was removed, and rats were allowed to swim freely for 120 s. Performance parameters included escape latency, swimming speed, and distance traveled during acquisition trials. During the probe trial, additional measurements included time spent in the target quadrant and the number of crossings over the previous platform location.

### 2.4 Sample Collection and Processing

Upon completion of the experimental protocol, the animals were anesthetized using sevoflurane (200 ppm, Sevorane; Queenborough, UK) and subsequently euthanized by decapitation. Brain tissues were harvested immediately post‐euthanasia. The extracted brain tissues were bifurcated, with one portion immediately cryopreserved at −80°C for subsequent biochemical assessments, and the other segment immersed in 10% neutral buffered formalin solution for histopathological evaluation.

All procedures were performed in strict accordance with institutional and international animal welfare guidelines (ARRIVE guidelines and European Directive 2010/63/EU). Throughout the experiment, animals were closely monitored for signs of neurotoxicity, pain, or distress. Anesthesia was administered prior to all invasive procedures to minimize suffering, and any animal exhibiting severe symptoms was promptly and humanely euthanized to prevent unnecessary distress. These measures ensured that animal welfare was prioritized at every stage of the study.

### 2.5 Lipid Peroxidation Analysis

Lipid peroxidation levels in brain tissues were determined by measuring the absorbance at 532 nm of the color released from the reaction between malondialdehyde (MDA) and thiobarbituric acid. For analysis, tissues were homogenized in a 1.15% potassium chloride (KCl) solution using a homogenizer. The homogenates were then centrifuged at 4°C and 1000 × g for 15 min, and the supernatant was used. The method developed by Placer et al. (1966) was employed to determine MDA levels.

### 2.6 Molecular Analysis of Gene Expression Through Real‐Time PCR Quantification

Transcriptional alterations in target genes (detailed in Table 1) were evaluated in brain tissue specimens using quantitative real‐time PCR methodology. Total RNA extraction was performed utilizing the QIAzol Lysis Reagent kit (79306; Qiagen) according to the manufacturer's specifications. Subsequently, complementary DNA (cDNA) was synthesized from the isolated RNA using the OneScript Plus cDNA Synthesis Kit (ABM, G236, Richmond, Canada). The PCR reaction mixtures were prepared by combining the synthesized cDNA templates with specific primer sequences and BlasTaq 2× qPCR MasterMix (ABM, G891, Richmond, Canada). Amplification was conducted using a Rotor‐Gene Q thermal cycler (Qiagen) following the manufacturer's recommended thermal cycling parameters. The relative quantification of gene expression was calculated using the comparative 2^−ΔΔCT^ method (Livak and Schmittgen 2001), with β‐Actin serving as the internal reference gene.

**TABLE 1:** Primer sequences.

Gene	Sequences (5′–3′)	Length (bp)	Accession no
CAT	F: ATGGCAACTGTCCCTGAACT R: AGTGACACTGCCTTCCTGAA	670	NM_012520.2
SOD	F: AGTCCCGCCCCTTCTAAAAC R: CAATGGCCTCTGTGTAGCCC	387	NM_001270850.1
GPx	F: CTCGAGTGACAAGCCCGTAG R: ATCTGCTGGTACCACCAGTT	290	NM_017006.2
Bcl‐2	F: GACTTTGCAGAGATGTCCAG R: TCAGGTACTCAGTCATCCAC	214	NM_016993.2
Cas‐3	F: ACTGGAATGTCAGCTCGCAA R: GCAGTAGTCGCCTCTGAAGA	270	NM_012922.2
Bax	F: TTTCATCCAGGATCGAGCAG R: AATCATCCTCTGCAGCTCCA	154	NM_017059.2
NF‐κB	F: AGTCCCGCCCCTTCTAAAAC R: CAATGGCCTCTGTGTAGCCC	106	NM_001276711.1
iNOS	F: AGATCAATGCAGCTGTGCTC R: GGCTCGATCTGGTAGTAGTAGA	235	NM_012611.3
TNF‐α	F: CTCGAGTGACAAGCCCGTAG R: ATCTGCTGGTACCACCAGTT	139	NM_012675.3
Beclin‐1	F: TCTCGTCAAGGCGTCACTTC R: CCATTCTTTAGGCCCCGACG	198	NM_053739.2
LC3A	F: GACCATGTTAACATGAGCGA R: CCTGTTCATAGATGTCAGCG	139	NM_199500.2
LC3B	F: GAGCTTCGAACAAAGAGTGG R: CGCTCATATTCACGTGATCA	152	NM_022867.2
Notch1	F: GTGGGATGGACTGGACTGTG R: GCGCAGGAAGTGGAAGGAGTT	117	NM_001105721
HES1	F: CGCCGGGCAAGAATAAATGA R: ATGTCTGCCTTCTCCAGCTT	104	NM_024360
β‐Actin	F: CAGCCTTCCTTCTTGGGTATG R: AGCTCAGTAACAGTCCGCCT	360	NM_031144.3

### 2.7 Histopathological and Immunohistochemical Evaluations

#### 2.7.1 Histopathological Evaluation

Brain specimens were fixed in neutral buffered formalin (10%) for a 72 h duration before undergoing routine tissue processing. The processed tissues were sectioned at 5 μm thickness and subjected to H&E staining for morphological assessment. Microscopic analysis was performed using an Olympus Cx43 microscope, with images captured via an EP50 digital camera system (Olympus Inc., Tokyo, Japan). Multiple fields were systematically evaluated for each specimen to assess tissue architecture and pathological changes. The assessment focused on key parameters including neuronal integrity, inflammatory response, cytoplasmic vacuolation, cell death patterns, and vascular alterations. A semi‐quantitative grading system was employed to categorize the severity of tissue changes (0: unremarkable, 1: minimal, 2: moderate, 3: marked).

#### 2.7.2 Immunohistochemical Assessment

Following routine processing, brain tissues were sectioned at a thickness of 4 μm from the cerebral cortex and hippocampus using a microtome for immunohistochemical analysis. The sections were deparaffinized in xylene and rehydrated through a graded alcohol series. Antigen retrieval was performed by treating the sections with citrate buffer at increasing temperatures in a microwave oven, followed by cooling. The sections were then washed with PBS and incubated in 3% H_2_O_2_ for 10 min to inactivate endogenous peroxidase activity. After washing with PBS, the tissue boundaries were outlined using a tissue boundary marker pen. The sections were blocked with the blocking solution from the Ultra Vision Detection System Large Volume Anti‐Polyvalent, HRP (RTU) kit (Thermo, TP‐125‐HP, UK) for 5 min. Subsequently, the sections were incubated overnight at +4°C with primary antibodies, including the apoptotic marker 8‐OHdG (sc‐66036, Santa Cruz Biotechnology, 1:100 dilution) and GFAP (sc‐33673, Santa Cruz Biotechnology, 1:100 dilution). After each step, the sections were washed with PBS and then incubated with the secondary antibody for 30 min, followed by treatment with streptavidin‐HRP (horseradish peroxidase) for 30 min. Next, 3,3′‐diaminobenzidine (DAB) was used as the chromogen, and counterstaining was performed with Harris hematoxylin. The results were evaluated under a light microscope based on the intensity of immunohistochemical brown staining. The immunohistochemical staining was graded on a scale of 0–3 for each section as follows: 0: no staining, 1: minimal staining, 2: moderate staining, and 3: intense staining. Five random sections from each group were selected, and staining intensity was analyzed using Image J software (Image J, version 1.46a, NIH, Bethesda, MD, USA).

### 2.8 Statistical Analyses

Quantitative data were processed using IBM SPSS statistical package. Numerical results are presented as mean values with standard deviation. Inter‐group comparisons were conducted using one‐way ANOVA with subsequent Tukey's post hoc analysis. Statistical significance was established at three threshold levels: *p* < 0.05, *p* < 0.01, and *p* < 0.001.

## 3 Results

### 3.1 Modulatory Effects of CRV on CP‐Mediated Oxidative Stress Parameters

**FIGURE 1:**
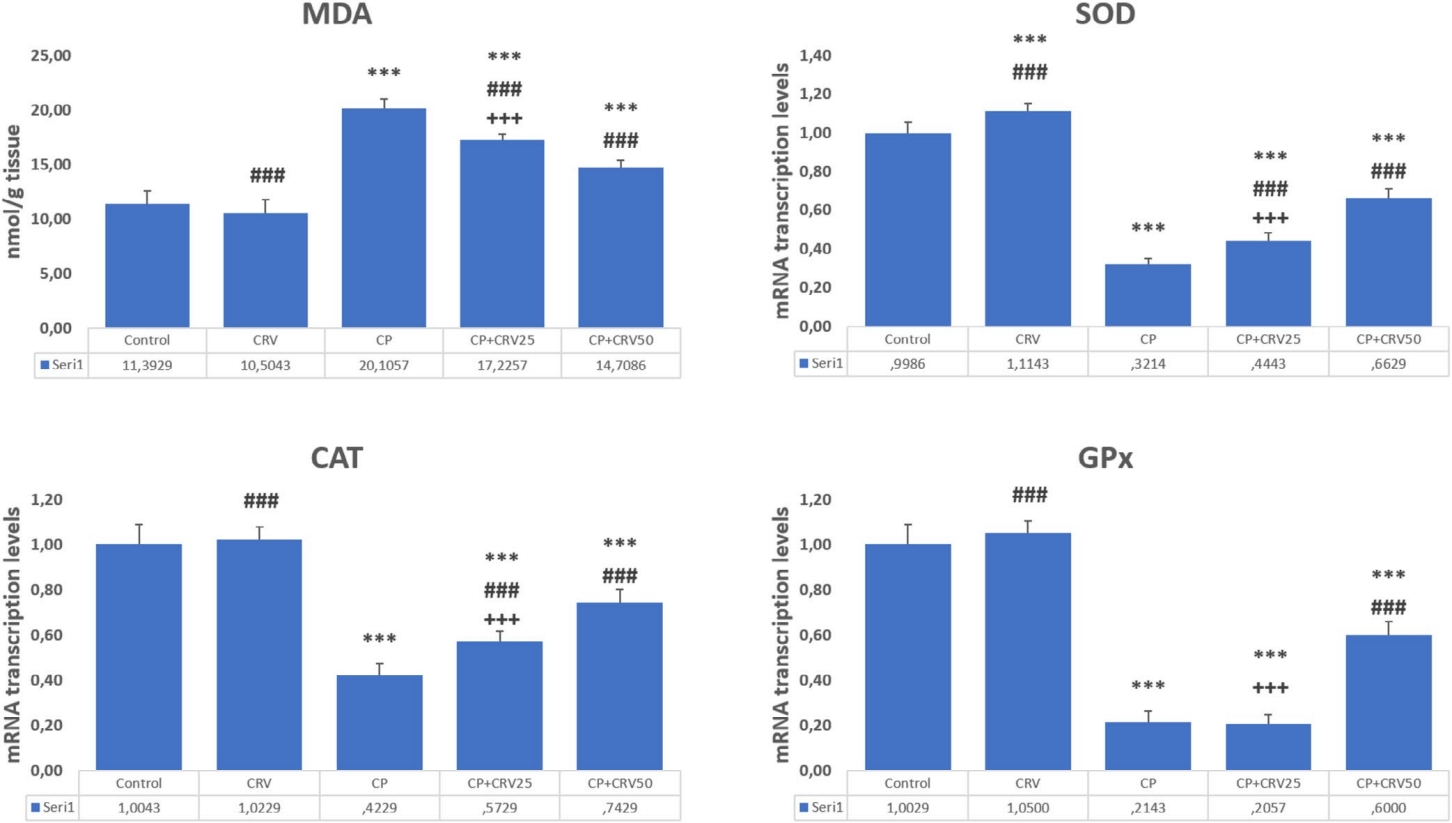
Effects of CRV on oxidative stress markers in CP‐induced neurotoxicity. Control versus others: *p < 0.05, **p < 0.01, ***p < 0.001, CP versus others: +*p* < 0.05, ++*p* < 0.01, +++*p* < 0.001, CP+ CRV25 versus CP+ CRV50: #*p* < 0.05, ##*p* < 0.01, ###*p* < 0.001 were analyzed using one‐way ANOVA, followed by Tukey's post hoc test.

Cyclophosphamide exposure induced significant oxidative stress, evidenced by markedly increased MDA levels (*p* < 0.001) and concurrent reduction in antioxidant enzyme activities (SOD, CAT, and GPx) relative to control animals (*p* < 0.001). CRV intervention, especially at the higher dose (50 mg/kg), successfully counteracted these biochemical alterations. Notably, the CP + CRV50 group exhibited superior protective effects, with more substantial normalization of lipid peroxidation markers (*p* < 0.001) and more effective restoration of enzymatic antioxidant defense systems (*p* < 0.001 for all measured parameters) when compared to the lower dose treatment group (Figure 1).

### 3.2 CRV Attenuates CP‐Induced Neuroinflammation Via NF‐κB/TNF‐α/iNOS Pathway

**FIGURE 2:**
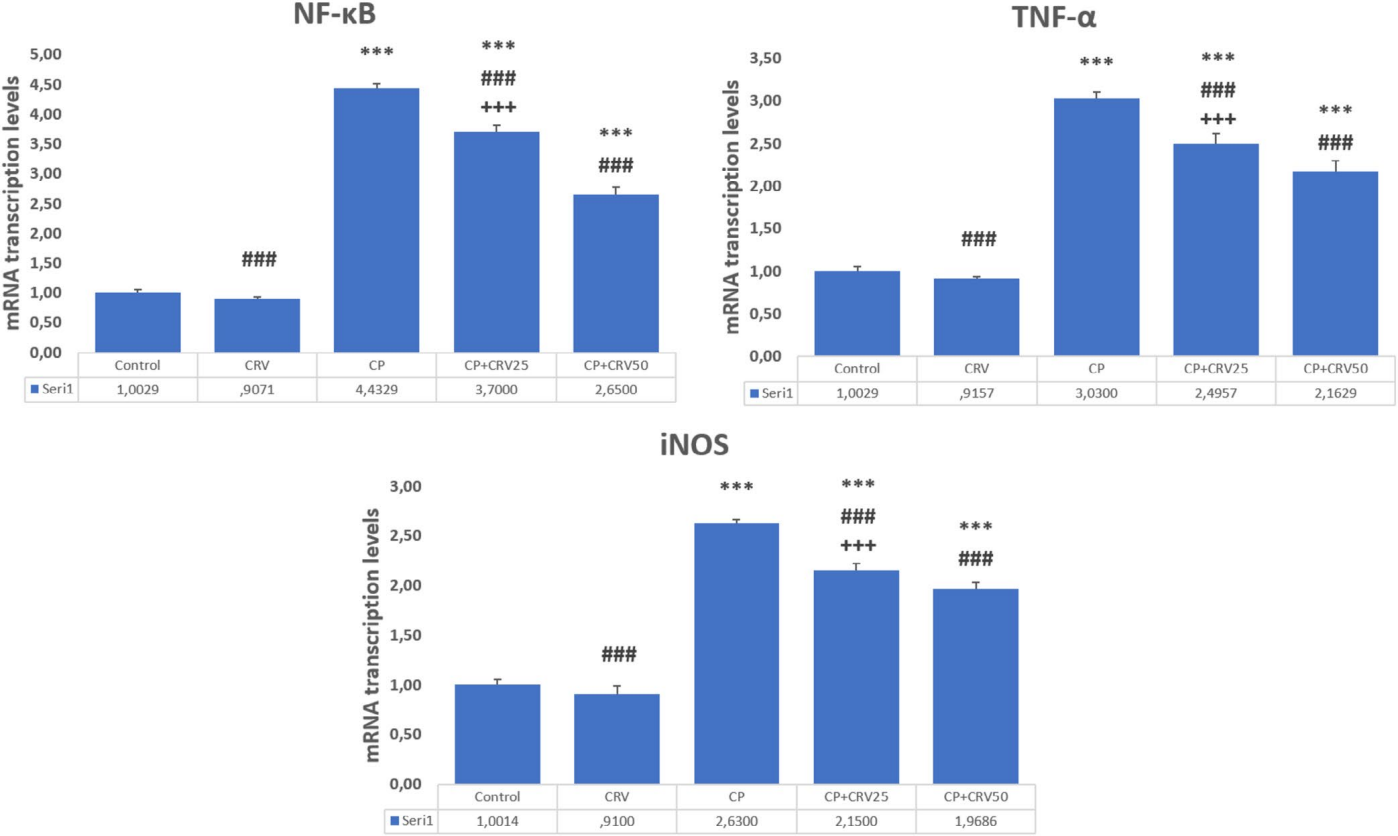
Effects of CRV on inflammatory markers in CP‐induced neurotoxicity. Control versus others: *p < 0.05, **p < 0.01, ***p < 0.001, CP versus others: +*p* < 0.05, ++*p* < 0.01, +++*p* < 0.001, CP + CRV25 versus CP + CRV50: #*p* < 0.05, ##*p* < 0.01, ###*p* < 0.001 were analyzed using one‐way ANOVA, followed by Tukey's post hoc test.

CP administration significantly upregulated inflammatory markers, with marked increases in NF‐κB (*p* < 0.001), TNF‐α (*p* < 0.001), and iNOS (*p* < 0.001) mRNA expression levels compared to the control group. CRV treatment effectively attenuated these inflammatory responses in a dose‐dependent manner. The CP + CRV50 group demonstrated more pronounced anti‐inflammatory effects, with significant reductions in NF‐κB (*p* < 0.001), TNF‐α (*p* < 0.001), and iNOS (*p* < 0.001) expression compared to the CP group. Additionally, the higher dose of CRV (50 mg/kg) showed superior anti‐inflammatory efficacy compared to the lower dose (25 mg/kg), as evidenced by the significant differences between the CP + CRV50 and CP + CRV25 groups (*p* < 0.001 for all inflammatory markers; Figure 2).

### 3.3 CRV Prevents CP‐Induced Neuronal Apoptosis by Regulating Casp‐3 Activation and Bax/Bcl‐2 Ratio

**FIGURE 3:**
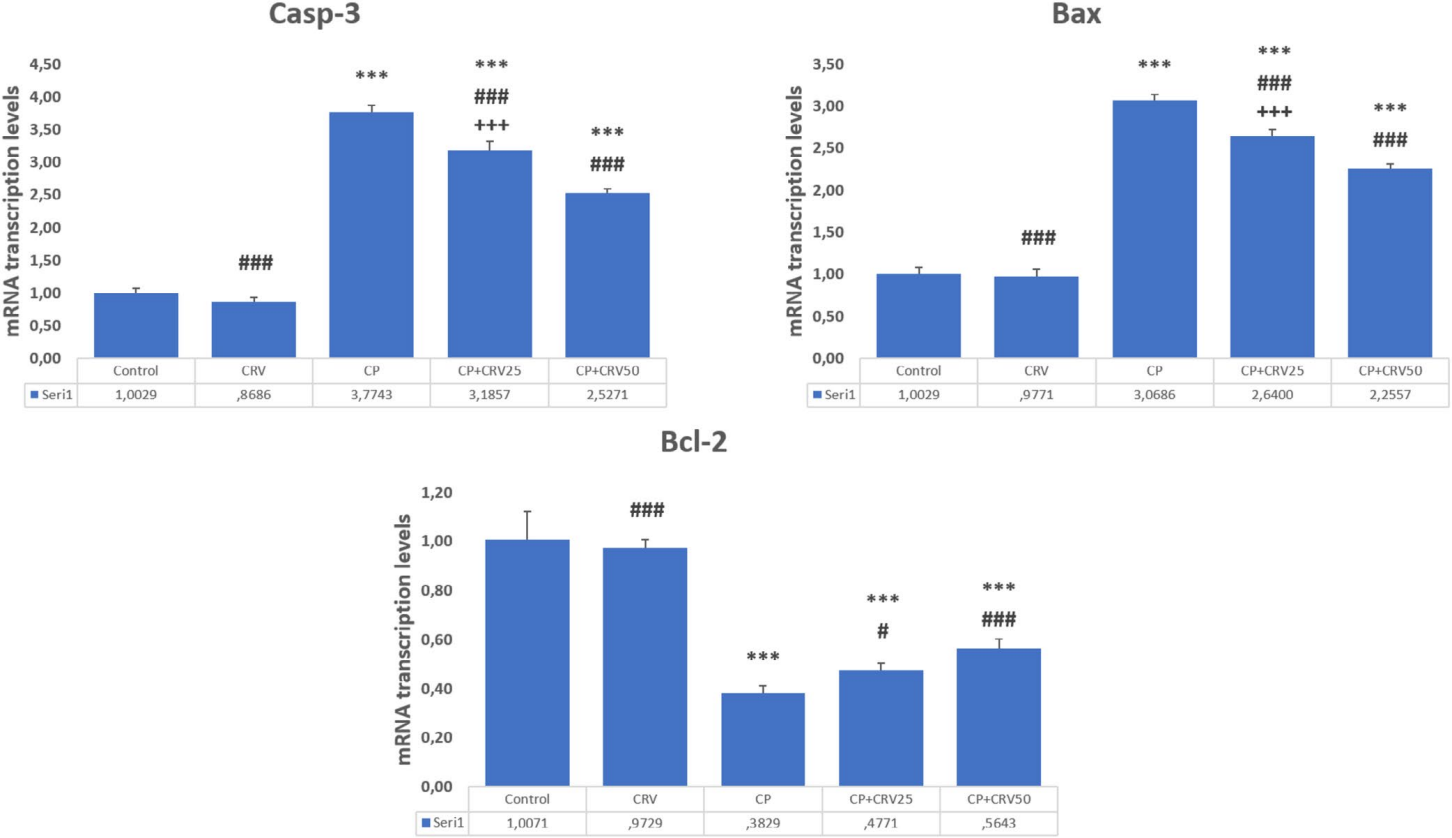
Effects of CRV on apoptotic markers in CP‐induced neurotoxicity. Control versus others: *p < 0.05, **p < 0.01, ***p < 0.001, CP versus others: +*p* < 0.05, ++*p* < 0.01, +++*p* < 0.001, CP + CRV25 versus CP + CRV50: #*p* < 0.05, ##*p* < 0.01, ###*p* < 0.001 were analyzed using one‐way ANOVA, followed by Tukey's post hoc test.

CP administration significantly disrupted apoptotic balance in brain tissue, as evidenced by marked upregulation of pro‐apoptotic markers Bax (*p* < 0.001) and Casp‐3 (*p* < 0.001), with concurrent downregulation of the anti‐apoptotic protein Bcl‐2 (*p* < 0.001) compared to the control group. CRV treatment effectively counteracted these alterations in a dose‐dependent manner. The CP + CRV50 group demonstrated superior anti‐apoptotic effects, with significant reduction in Bax (*p* < 0.001) and Casp‐3 (*p* < 0.001) expression, alongside enhanced Bcl‐2 levels (*p* < 0.001) compared to the CP group. Furthermore, the higher dose of CRV (50 mg/kg) exhibited more potent anti‐apoptotic activity than the lower dose (25 mg/kg), as indicated by significant differences between the CP + CRV50 and CP + CRV25 groups (*p* < 0.001 for all apoptotic markers; Figure 3).

### 3.4 Protective Effects of CRV Against CP‐Induced Dysregulation of Autophagic Flux

**FIGURE 4:**
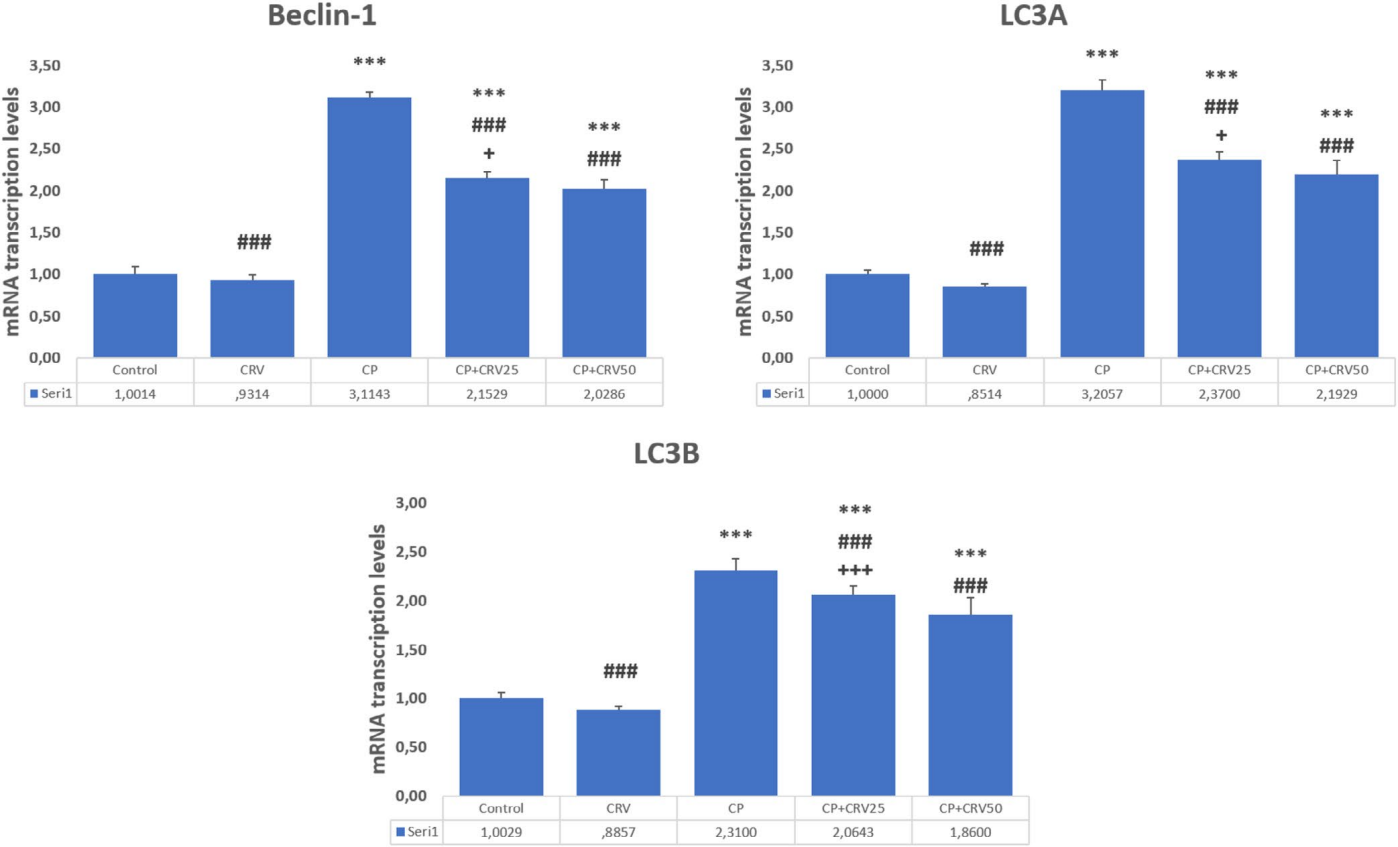
Effects of CRV on autophagy markers in CP‐induced neurotoxicity. Control versus others: *p < 0.05, **p < 0.01, ***p < 0.001, CP versus others: +*p* < 0.05, ++*p* < 0.01, +++*p* < 0.001, CP + CRV25 versus CP + CRV50: #*p* < 0.05, ##*p* < 0.01, ###*p* < 0.001 were analyzed using one‐way ANOVA, followed by Tukey's post hoc test.

CP administration significantly dysregulated autophagic processes in brain tissue, as demonstrated by marked upregulation of autophagy markers Beclin‐1 (*p* < 0.001), LC3A (*p* < 0.001), and LC3B (*p* < 0.001) compared to the control group. CRV treatment effectively normalized these alterations in a dose‐dependent manner. The CP + CRV50 group exhibited superior regulatory effects on autophagy, with significant reduction in Beclin‐1 (*p* < 0.001), LC3A (*p* < 0.001), and LC3B (*p* < 0.001) expression compared to the CP group. Additionally, the higher dose of CRV (50 mg/kg) demonstrated more effective modulation of autophagic flux than the lower dose (25 mg/kg), as evidenced by significant differences between CP + CRV50 and CP + CRV25 groups (*p* < 0.001 for Beclin‐1 and LC3B, *p* < 0.05 for LC3A; Figure 4).

### 3.5 CRV Preserves Neuronal Integrity by Restoring CP‐Impaired Notch1/Hes1 Signaling Pathway

**FIGURE 5:**
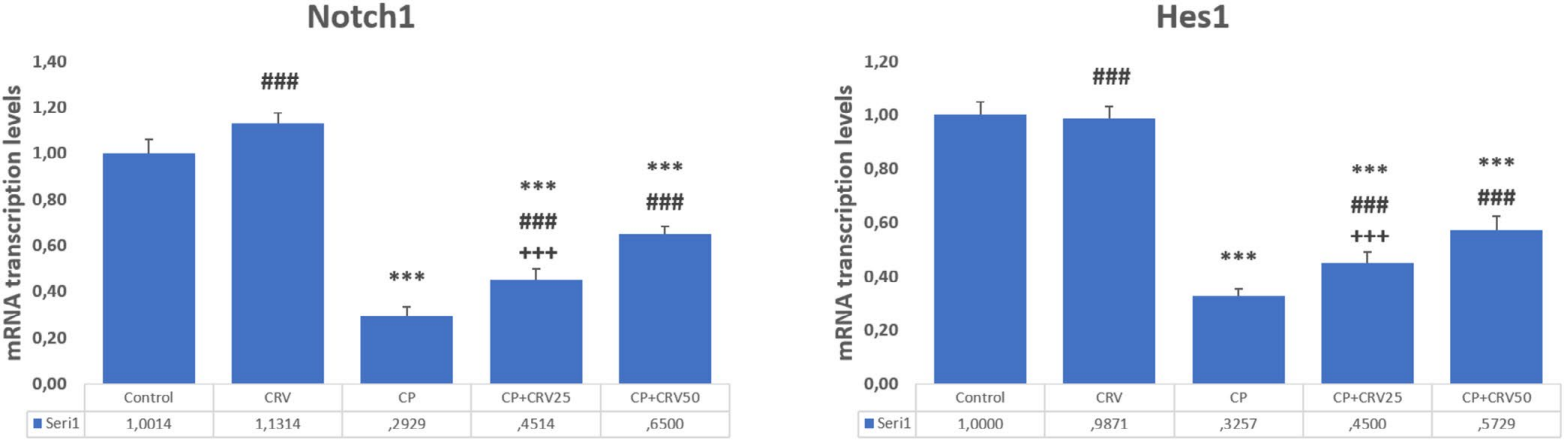
Effects of CRV on Notch1/Hes1 Signaling Pathway in CP‐induced neurotoxicity. Control versus others: *p < 0.05, **p < 0.01, ***p < 0.001, CP versus others: +*p* < 0.05, ++*p* < 0.01, +++*p* < 0.001, CP + CRV25 versus CP + CRV50: #*p* < 0.05, ##*p* < 0.01, *###p* < 0.001 were analyzed using one‐way ANOVA, followed by Tukey's post hoc test.

CP administration significantly suppressed Notch signaling in brain tissue, as evidenced by marked downregulation of both Notch1 (*p* < 0.001) and its downstream effector Hes1 (*p* < 0.001) compared to the control group. CRV treatment effectively restored this critical neuroprotective pathway in a dose‐dependent manner. The CP + CRV50 group demonstrated superior restorative effects, with significant enhancement of Notch1 (*p* < 0.001) and Hes1 (*p* < 0.001) expression compared to the CP group. Furthermore, the higher dose of CRV (50 mg/kg) exhibited more potent activity in preserving Notch signaling than the lower dose (25 mg/kg), as indicated by significant differences between the CP + CRV50 and CP + CRV25 groups (*p* < 0.001 for both Notch1 and Hes1; Figure 5).

### 3.6 Histopathological and Immunohistochemical Evidence of CRV‐Mediated Neuroprotection

#### 3.6.1 Effect of CP and CRV on Cerebral Cortex and Hippocampus Morphology

**FIGURE 6:**
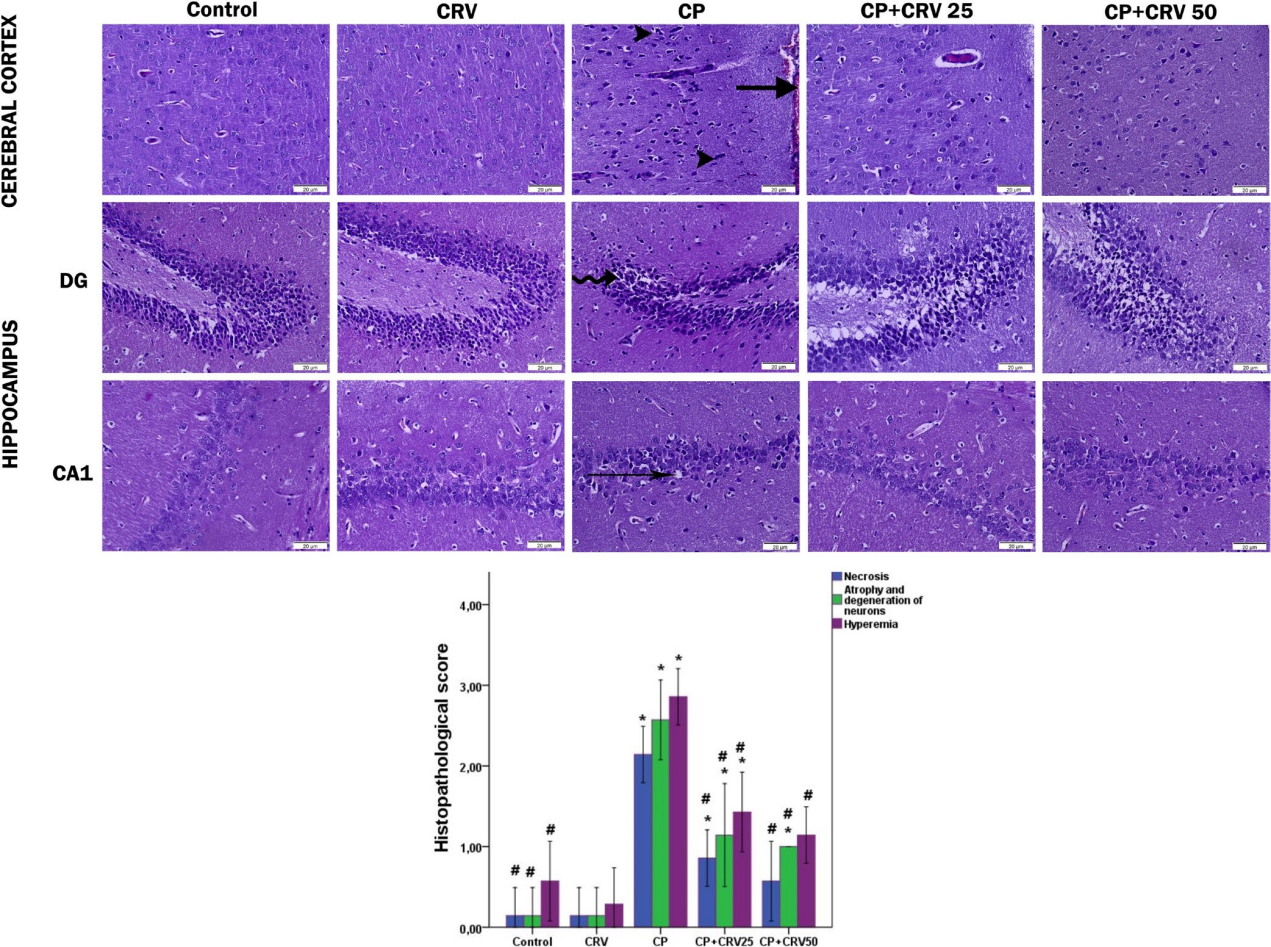
Photomicrographs of the cerebral cortex and hippocampal regions, including the dentate gyrus (DG) and cornu ammonis 1 (CA1), stained with H&E for the control and different experimental groups (×400). The cerebral cortex and hippocampal tissues of the control and CRV groups exhibited normal structures. The CP group showed an increase in degenerative cells (arrowhead) and hyperemia (thick arrow), as well as deeply dark pyknotic nuclei (curved arrow) and pericellular halos (thin arrow) in the DG and CA1 regions. The CP + CRV 25 and CP + CRV 50 groups demonstrated recovery resembling the control group, except for a reduction in degenerative cells. Control versus others: *p < 0.05, CP versus others: #*p* < 0.05, CP + CRV25 versus CP + CRV50: +*p* < 0.05.

The hematoxylin–eosin staining results and histopathological score graph of the cerebral cortex and hippocampal regions, including the cornu ammonis (CA) and dentate gyrus (DG), for the control and different groups are presented in Figure 6. Histological observations revealed that the cerebral cortex and hippocampal sections of the control and CRV‐treated groups exhibited normal histological structures. In the micrographs of the CP group, atrophic changes in localized neurons of the cerebral cortex and degeneration in neurons with pyknotic nuclei were prominent. Additionally, the meningeal vessels were excessively hyperemic. The hippocampal sections of this group showed intense pyknotic changes, and some neurons and glial cells were surrounded by pericellular halos. In the groups treated with CRV alongside CP, the pathological features in the cerebral cortex were reduced, and the histological characteristics resembled those of the control sections. Only rare areas showed neuronal degeneration and vascular hyperemia. According to the histopathological scoring results in the graph shown in Figure 6, the CP group exhibited a significantly higher histopathological damage score compared to the control group (*p* < 0.05). However, in the treatment groups where CRV was administered following CP, the damage score was significantly reduced compared to the CP group (*p* < 0.05).

#### 3.6.2 Effect of CP and CRV on GFAP and 8‐OHdG Immunostaining in the Cerebral Cortex and Hippocampus

**FIGURE 7:**
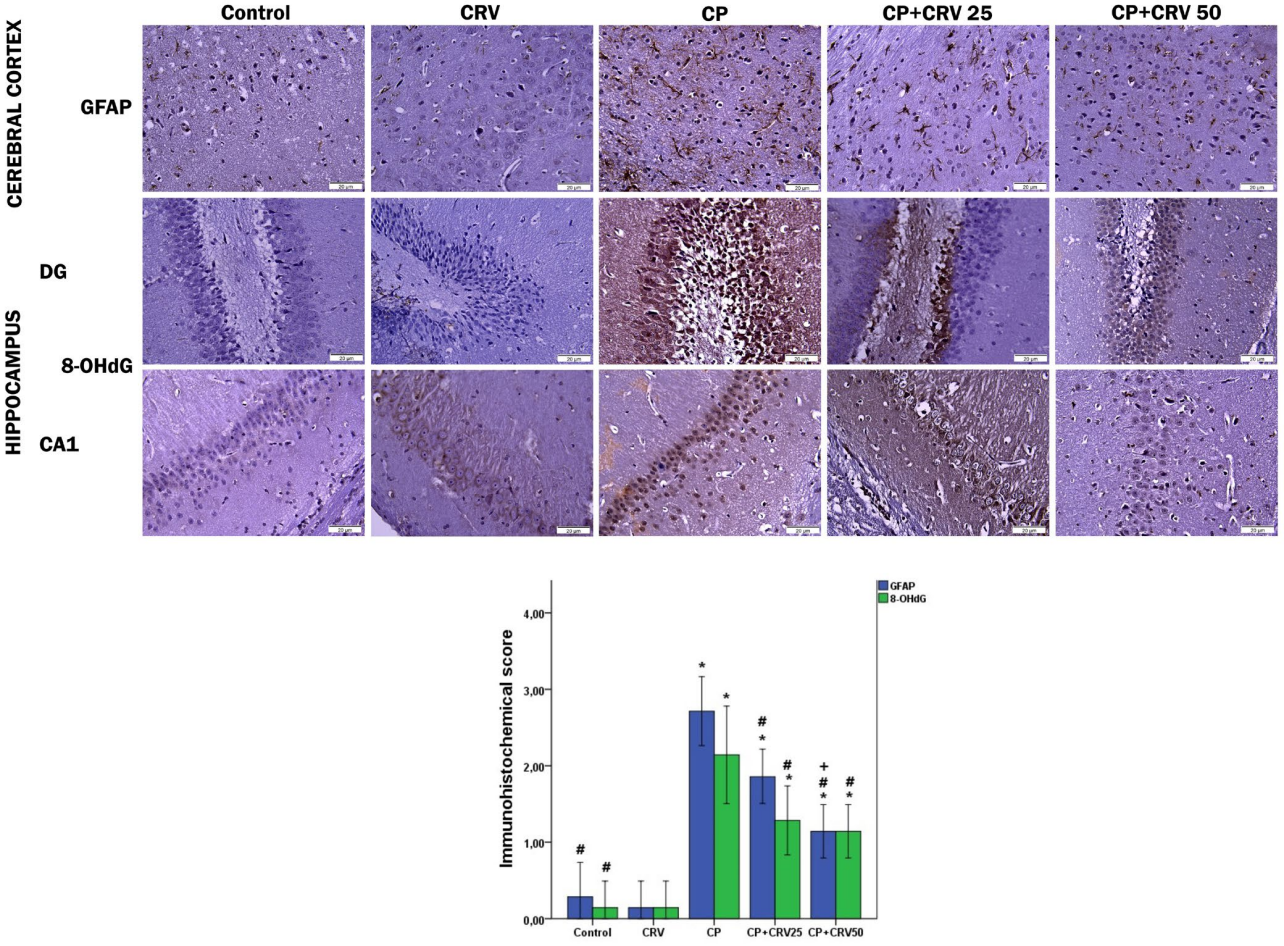
Photomicrographs of the cerebral cortex and hippocampal regions, including the dentate gyrus (DG) and cornu ammonis 1 (CA1), stained with immunohistochemical markers for the control and different experimental groups (×400). The cerebral cortex and hippocampal tissues of the control and CRV groups showed mild GFAP and 8‐OHdG expression. In the CP group, there was a marked increase in GFAP expression in the cerebral cortex and 8‐OHdG expression in the hippocampus. In the CP + CRV 25 and CP + CRV 50 groups, a reduction in GFAP and 8‐OHdG expression in the cerebral cortex and 8‐OHdG expression in the hippocampus was observed. Control versus others: *p < 0.05, CP versus others: #*p* < 0.05, CP + CRV 25 versus CP + CRV 50: +*p* < 0.05.

The results of GFAP and 8‐OHdG immunohistochemical staining and evaluation in brain tissue are presented in Figure 7. In the control and CRV groups, GFAP and 8‐OHdG immunopositivity in the cerebral cortex and hippocampus was very mild. In the CP group, GFAP immunoexpression in the cerebral cortex of the rats was severe. Additionally, in the same group, there was a significant increase in immunopositivity in the hippocampus due to the elevated levels of 8‐OHdG protein. However, in the CP + CRV25 and CP + CRV50 groups, the expression of GFAP and 8‐OHdG was significantly reduced compared to the CP group (*p* < 0.05).

### 3.7 Effects of CRV on CP‐Induced Cognitive Parameters in Morris Water Maze Test

**FIGURE 8:**
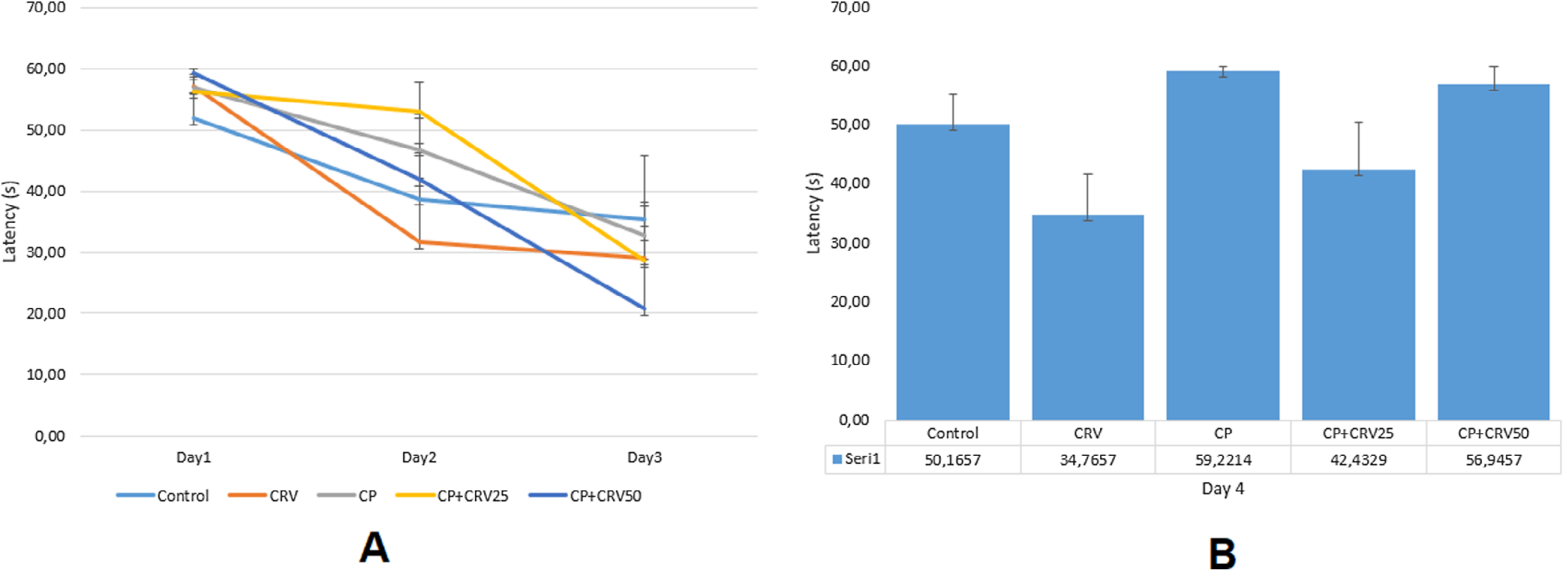
Effects of CRV on spatial learning in CP‐induced neurotoxicity as assessed by Morris Water Maze test. (A) Comparison of escape latency across groups; (B) Changes in escape latency over training days (days 1–4). CP administration showed a tendency to increase escape latency, while CRV treatment demonstrated a dose‐dependent trend toward improvement in this parameter. However, no statistically significant differences were detected between groups (*p* > 0.05). Data were analyzed using one‐way ANOVA, followed by Tukey's post hoc test.

Morris Water Maze test was employed to evaluate the effects of CP and CRV on spatial learning and memory functions. The results revealed trends consistent with expected cognitive effects, though without reaching statistical significance. In the escape latency assessment (Figure 8A), CP‐treated rats showed a noticeable increase (approximately 40% higher than control), suggesting potential learning impairment, while both CRV treatment groups demonstrated dose‐dependent decreases in escape latency, with CP + CRV50 group values approaching control levels. The acquisition curve over training days (Figure 8B) showed that all groups tended to improve their performance across days, with the CP group consistently displaying longer latencies compared to other groups.

**FIGURE 9:**
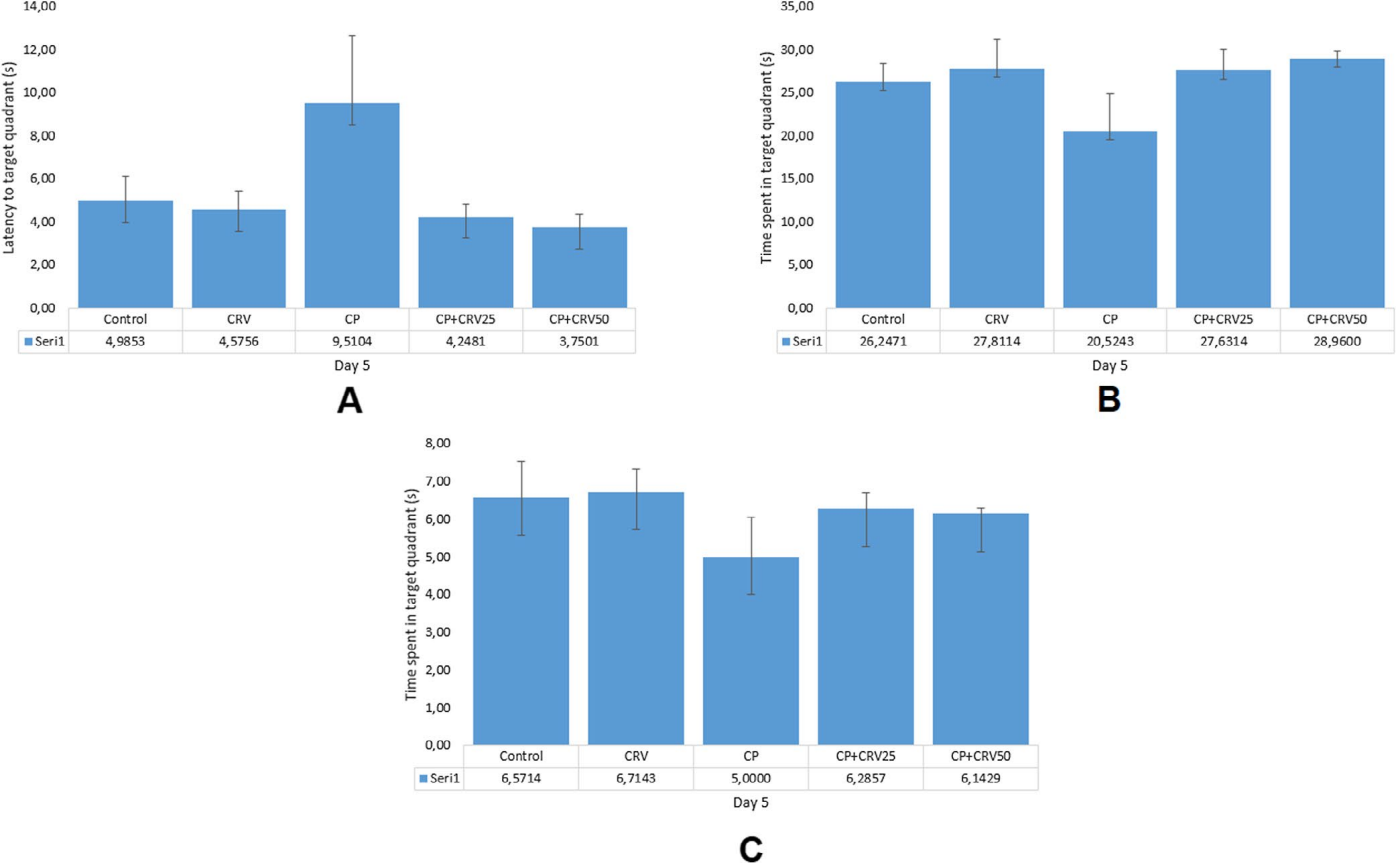
Effects of CRV on spatial memory in CP‐induced neurotoxicity as assessed by the Morris water maze probe trial. (A) Latency to target quadrant; (B) Time spent in target quadrant; (C) Time spent in target quadrant (repeated assessment). CP administration showed a tendency to decrease time spent in the target quadrant and increase latency to the target quadrant, while CRV treatment demonstrated a dose‐dependent trend toward improvement in these parameters. However, statistical analysis revealed no significant differences between the groups (*p* > 0.05).

In the probe trial, latency to target quadrant (Figure 9B) was elevated in the CP group compared to control, with apparent reductions in both CRV treatment groups. Time spent in target quadrant (Figure 9A), a key indicator of spatial memory retention, showed the expected pattern with CP administration resulting in decreased time (approximately 50% reduction compared to control), while CRV treatment appeared to dose‐dependently counteract this effect, with the CP + CRV50 group showing values closest to control levels. These observed trends align with the anticipated neuroprotective effects of CRV against CP‐induced cognitive impairment, despite the lack of statistical significance. The consistent pattern across multiple parameters suggests a potential biological effect that warrants further investigation with refined methodologies or increased sample sizes to achieve statistical power.

## 4 Discussion

Carvacrol (CRV), a natural monoterpenic phenol found in various aromatic plants, has attracted increasing scientific interest due to its well‐documented antioxidant, anti‐inflammatory, and neuroprotective properties (Ali et al. 2024). Recent studies have suggested that CRV may modulate several cellular pathways involved in neuronal injury. Building on this evidence, the present study aimed to investigate the potential effects of CRV in a model of chemotherapy‐induced neurotoxicity.

Chemotherapy‐induced neurotoxicity, particularly associated with widely used agents such as cyclophosphamide (CP), remains a significant clinical problem in cancer treatment (Ibrahim et al. 2024; Yu et al. 2024). Despite the therapeutic efficacy of CP, its neurotoxic effects can adversely affect patients' quality of life and complicate adherence to treatment (Taillibert et al. 2016). The pathophysiology of CP‐induced neurotoxicity involves the interplay of multiple cellular mechanisms, including oxidative stress, neuroinflammation, and apoptosis (Ren et al. 2019). The inadequacy of current treatment options highlights the need for new approaches in this field (Ali et al. 2024). Natural antioxidant and anti‐inflammatory compounds are being investigated for this purpose (Mohd Sairazi and Sirajudeen 2020). Therefore, in this study, the effects of carvacrol (CRV) on CP‐induced neurotoxicity were examined at the level of multiple signaling pathways.

The brain's vulnerability to oxidative damage is associated with its high oxygen consumption, rich polyunsaturated fatty acid content, and limited antioxidant defense capacity (Halliwell 2006; Sultana et al. 2025). These features render the brain particularly susceptible to the harmful effects of oxidative stress (Garbarino et al. 2015; Lee et al. 2020). Oxidative stress can impair neuronal function by weakening antioxidant defense mechanisms and triggering various cellular stress responses such as apoptosis, inflammatory cascades, and endoplasmic reticulum stress (Gao et al. 2018). CP exerts its neurotoxic effects through metabolic activation, leading to the formation of toxic metabolites and depletion of cellular antioxidant reserves (Famurewa et al. 2023; Singh and Kumar 2019).

The primary defense against oxidative damage in the brain consists of antioxidant systems such as superoxide dismutase (SOD), catalase (CAT), glutathione peroxidase (GPx), and the key cofactor glutathione (GSH) (Dringen et al. 2005). These systems work together: SOD converts superoxide anions to hydrogen peroxide (Fukai and Ushio‐Fukai 2011), and CAT breaks down hydrogen peroxide into water and molecular oxygen, thus preventing the formation of harmful hydroxyl radicals (Ighodaro and Akinloye 2018). The glutathione system plays a critical role in maintaining cellular redox balance by reducing various organic hydroperoxides (Averill‐Bates 2023; He et al. 2017). Malondialdehyde (MDA), the main end product of lipid peroxidation, is an important indicator of oxidative damage (Yekti et al. 2018), and high levels reflect cellular membrane disruption and neuronal dysfunction (Ma et al. 2025).

In our study, CP administration resulted in marked oxidative stress, as evidenced by increased MDA levels and decreased activities of antioxidant enzymes such as SOD, CAT, and GPx. CRV treatment provided significant improvement in these parameters compared to the CP group. Similarly, normalization was observed in the mRNA expression levels of antioxidant enzymes. These findings suggest that CRV may exert a regulatory effect on oxidative stress parameters. It is known that the oxidative stress response is regulated via the Nrf2 (nuclear factor erythroid 2‐related factor 2) signaling pathway.

CP‐induced neuroinflammation is characterized by increased expression of pro‐inflammatory cytokines and enzymes (NF‐κB, TNF‐α, iNOS; Famurewa et al. 2021; Gupta et al. 2022). In our study, CP administration significantly increased the expression of these inflammatory markers, while CRV treatment reduced these increases in a dose‐dependent manner. Inflammation is thought to play a role in the progression of neuronal damage, together with oxidative stress (Ibrahim et al. 2024). The inflammatory cascade initiated by CP involves the activation of various pro‐inflammatory mediators such as TNF‐α, contributing to neuronal damage and dysfunction. This neuroinflammatory response is closely linked to oxidative stress and may create a self‐perpetuating cycle of cellular injury (Akaras, Kucukler, et al. 2024; Elsayed et al. 2022). The process mainly occurs through the activation of the NF‐κB signaling pathway, where increased oxidative stress leads to nuclear translocation of NF‐κB dimers. Nuclear NF‐κB initiates the expression of pro‐inflammatory cytokines and enzymes, especially TNF‐α and inducible nitric oxide synthase (iNOS). In conditions such as acute trauma, stress (Tanyeli et al. 2020), or inflammation, high concentrations of nitric oxide produced by iNOS can have both protective and harmful effects on tissues; excessive production generally increases tissue damage (Benzer, Kandemir, Kucukler, et al. 2018; Ileriturk et al. 2024).

In the present study, CP administration led to significant increases in inflammatory markers, while CRV treatment reduced these increases in a dose‐dependent manner. Moreover, high‐dose CRV (50 mg/kg) showed a more pronounced anti‐inflammatory effect compared to the lower dose (25 mg/kg). The effects of inflammation on neural tissue may contribute to the progression of cellular injury and the development of chronic pathological processes in the brain (DiSabato et al. 2016; Lyman et al. 2014). It is known that CP exacerbates the inflammatory response, leading to neuronal damage in brain tissue and further impairing brain function (Mishra et al. 2022; Taslimi et al. 2019). In the present study, CRV effectively suppressed the inflammatory response by reducing pro‐inflammatory cytokine expression and preventing inflammatory cell infiltration, thereby preserving neuronal integrity in brain tissue.

Apoptosis is another important component of CP‐induced neurotoxicity. In our findings, CP administration resulted in increased expression of Bax and Casp‐3 and decreased Bcl‐2 expression. CRV treatment normalized these parameters compared to the CP group. The changes in apoptotic markers paralleled those observed in inflammation and oxidative stress parameters. Mitochondria‐mediated apoptotic signaling is a key mechanism underlying CP‐induced neurodegeneration and functions as a finely regulated cell death cascade involving the interaction between pro‐apoptotic and anti‐apoptotic mediators (Ibrahim et al. 2024). Mitochondrial membrane integrity is determined by the dynamic balance between the pro‐apoptotic protein Bax, which facilitates membrane permeability, and the anti‐apoptotic protein Bcl‐2, which maintains membrane stability (Suhaili et al. 2017; Şimşek et al. 2024). Following mitochondrial membrane disruption, the release of cytochrome c initiates the sequential activation of Casp‐3, the main executioner protease responsible for cellular destruction and chromatin fragmentation (Bal et al. 2023). Disruptions in this tightly regulated pathway can lead to abnormal activation of apoptosis, reduced neuronal viability, and impaired cerebral function (Bazhanova and Kozlov 2024). In our study, CRV treatment normalized these parameters compared to the CP group.

Autophagy, a highly conserved catabolic mechanism, involves the degradation and recycling of cellular components through the formation of autophagosomes and their subsequent fusion with lysosomes, serving dual functions in neuronal health by either promoting cell survival through the elimination of damaged organelles and protein aggregates or contributing to cell death when excessively activated (Parzych and Klionsky 2014). Autophagy was evaluated by the increased expression of Beclin‐1, LC3A, and LC3B following CP administration. CRV treatment reduced these markers compared to the CP group. The changes in autophagy parameters were observed in parallel with those in apoptosis and Notch1/Hes1 signaling (Valencia et al. 2021). Autophagy is an important process for maintaining cellular homeostasis and responding to stress, and a marked increase in this process was observed with CP administration. CRV treatment reduced this increase. The observation that changes in autophagy markers occurred together with changes in apoptosis and Notch1/Hes1 signaling suggests that there may be a relationship between these pathways (de la Hoz‐Camacho et al. 2022; Ibrahim et al. 2024).

The Notch1/Hes1 signaling pathway is an important mechanism involved in neuronal survival and cellular homeostasis (Ables et al. 2011; Ma et al. 2025). CP administration resulted in decreased expression of Notch1 and Hes1. CRV treatment increased these parameters compared to the CP group. The observation that changes in the Notch1/Hes1 pathway occurred together with changes in autophagy and apoptosis markers suggests that there may be a relationship between these pathways (Li et al. 2023). The Notch signaling pathway, a highly conserved cell‐to‐cell communication system, plays pivotal roles in neural development, adult neurogenesis, and neuronal survival, and is initiated by the interaction between Notch receptors (particularly Notch1) and their ligands, leading to a series of proteolytic cleavages that release the Notch intracellular domain (NICD) The NICD subsequently translocates to the nucleus, where it regulates the expression of target genes, including the Hes1 (Hairy and enhancer of split‐1) transcription factor (Akaras, Kandemir, and Şimşek 2024). Hes1, in turn, influences various aspects of neural function, including cell fate decisions, differentiation, and survival (Niaz et al. 2025). The Notch1/Hes1 pathway emerges as a critical regulatory axis in neurodegenerative processes, serving as a central mediator in maintaining neuronal homeostasis, protecting against oxidative stress, and regulating autophagy mechanisms (Ma et al. 2025). Recent evidence suggests that Notch signaling modulates the delicate balance between neuronal survival and death pathways, particularly under conditions of cellular stress. Furthermore, Notch1 activation has been shown to enhance neuroprotective mechanisms by promoting anti‐apoptotic gene expression and supporting mitochondrial function (Wang et al. 2022). Recent investigation into the Notch1/Hes1 pathway revealed significant alterations following CP administration. CP administration significantly suppressed Notch signaling in brain tissue, as evidenced by marked downregulation of both Notch1 (*p* < 0.001) and its downstream effector Hes1 (*p* < 0.001) compared to the control group. This suppression likely represents a key molecular mechanism underlying CP‐induced neurotoxicity, as diminished Notch1/Hes1 signaling may compromise neuronal survival mechanisms, impair neural regenerative capacity, and disrupt the coordinated regulation of autophagy. The relationship between Notch1/Hes1 downregulation and the observed increases in oxidative stress, neuroinflammation, and apoptotic markers suggests that this pathway serves as an integrative hub that influences multiple aspects of neuronal health and function under chemotherapy‐induced stress conditions.

In the present study, most of the changes observed in oxidative stress, inflammation, apoptosis, autophagy, and Notch1/Hes1 signaling occurred with CP administration and were partially or completely normalized with CRV treatment. These findings suggest that CP‐induced neurotoxicity arises from the interaction of multiple cellular processes and that CRV may exert regulatory effects on these processes. However, further studies are needed to clarify the causal relationships between these pathways and the direct effects of CRV on these processes (Li et al. 2023).

Histopathological and immunohistochemical analyses showed increased expression of GFAP and 8‐OHdG with CP administration, which was reduced by CRV treatment. GFAP is a well‐known marker of astrogliosis and neuroinflammation and is a sensitive indicator of neuronal injury and reactive astrocyte activation (O'Callaghan and Sriram 2005; Yardim et al. 2020). CP administration significantly increased GFAP immunoreactivity in the cerebral cortex, indicating marked astrocytic activation in response to neurotoxic injury. While increased GFAP expression generally reflects a defense mechanism against brain tissue injury, persistent astrogliosis may exacerbate neuroinflammation and contribute to neurodegeneration (Hegab et al. 2025; O'Callaghan 1988). At the same time, 8‐OHdG, a reliable marker of oxidative DNA damage, was markedly increased in the hippocampus of CP‐treated rats, providing direct evidence of nucleic acid modifications due to oxidative stress (Benzer, Kandemir, Ozkaraca, et al. 2018; Patwa et al. 2020). The presence of elevated 8‐OHdG, especially indicating guanine oxidation in DNA, can impair neuronal function and viability as a critical biomarker of oxidative stress‐mediated cellular injury (Caglayan et al. 2019; Piao et al. 2005). CRV treatment reduced both GFAP and 8‐OHdG immunoreactivity in a dose‐dependent manner, with the higher dose (50 mg/kg) showing greater efficacy. These immunohistochemical findings are consistent with our molecular data showing the suppressive effects of CRV on inflammatory mediators (NF‐κB/TNF‐α/iNOS) and oxidative stress parameters, suggesting coordinated protection across multiple cellular mechanisms.

In behavioral assessments, CP administration resulted in impairment of spatial learning and memory functions, while CRV treatment partially corrected this impairment. In the escape latency assessment, rats treated with CP showed a marked increase (about 40% higher than controls), indicating possible learning deficits. Both CRV treatment groups showed dose‐dependent decreases in escape latency, with the CP + CRV50 group approaching control values. During the acquisition curve over training days, all groups tended to improve their performance over time, but the CP group consistently exhibited longer latencies than the other groups. In the probe test, the time spent in the target quadrant (the main indicator of spatial memory retention) decreased as expected with CP administration (about 50% reduction compared to controls), while CRV treatment reversed this effect in a dose‐dependent manner, with the CP + CRV50 group being closest to control values. These observed trends are consistent with the expected neuroprotective effects of CRV against CP‐induced cognitive impairment. When these behavioral trends are evaluated together with molecular findings, they provide additional support for the neuroprotective potential of CRV against CP‐induced cognitive dysfunction.

## 5 Conclusions

In conclusion, this study demonstrated that CRV administration in a CP‐induced neurotoxicity model may exert regulatory effects on multiple cellular processes, including oxidative stress, inflammation, apoptosis, autophagy, and the Notch1/Hes1 signaling pathway. Notably, the neuroprotective effects of CRV were dose‐dependent, with 50 mg/kg providing superior protection compared to 25 mg/kg. By modulating these interconnected stress response pathways, CRV shows significant therapeutic potential in addressing chemotherapy‐induced neurotoxicity. These findings highlight CRV as a promising natural compound for the development of targeted neuroprotective strategies and underscore the need for further clinical investigation to more precisely elucidate the relationships between these pathways and the effects of CRV.

## Author Contributions

**Hamit Emre Kızıl:** conceptualization, experimental design, investigation, data collection, formal analysis, methodology, visualization, writing – original draft.

## Ethics Statement

Ethics committee approval was received for this study from the ethics committee of Necmettin Erbakan University KONUDAM Experimental Medicine Research and Study Centers (No: 2025‐10; Date: February 20, 2025). All experiments were performed in accordance with the European Directive 2010/63/EU for animal experiments. In addition, all animal‐related procedures used in this study were performed in accordance with ARRIVE guidelines.

## Consent

The author has nothing to report.

## Conflicts of Interest

The author declares no conflicts of interest.

## Data Availability

All data generated or analyzed during this study are included in this published article.
